# Efficacy of simultaneous aerobic and cognitive training (Activ4Brain) on physical fitness and body composition in older persons

**DOI:** 10.3389/fragi.2026.1819235

**Published:** 2026-06-09

**Authors:** Kaan Akalp, Hicran Besikci, Afonso Rainho Mateus, Luís Rama, José Pedro Ferreira, Maria José Ribeiro, Ana Maria Teixeira

**Affiliations:** 1 Faculty of Sport Sciences and Physical Education, University of Coimbra, Coimbra, Portugal; 2 CIPER- Research Center for the Study of Human Performance, University of Coimbra, Coimbra, Portugal; 3 CIBIT-ICNAS, University of Coimbra, Coimbra, Portugal; 4 Faculty of Medicine, University of Coimbra, Coimbra, Portugal; 5 RISE-Health, Center for Translational Health and Medical Biotechnology (TBIO), School of Health (ESS), Polytechnic of Porto, Porto, Portugal

**Keywords:** aerobic exercise, aging, fat to muscle ratio, muscle strength, phase angle

## Abstract

**Introduction:**

The popularity of simultaneously integrated cognitive training and exercise programs is increasingly adopted as a strategy to mitigate age-related declines in cognitive function and physical fitness, and these benefits may be further enhanced in a social context. However, the effects of integrated programs particularly when delivered as group activities on physical fitness and body composition have not been comprehensively investigated. Therefore, the purpose of this study is to evaluate and compare the effects of the Activ4Brain program on physical fitness and body composition with those of aerobic exercise alone.

**Methods:**

Seventy-four cognitively healthy older persons, with an average age of 65.5 ± 6.2 years, were assigned to control (n = 27) aerobic exercise (AE) (n = 23) and integrated cognitive and aerobic exercise (Activ4Brain) groups (n = 24). Physical fitness was assessed using a cardiopulmonary exercise test (CPET), timed up and go (TUG), 30 s chair stand, and hand grip strength tests. Body composition was assessed using bioimpedance (Inbody 770, Biospace). Evaluated parameters included body mass index (BMI), fat mass, fat percentage, muscle mass, fat to muscle ratio (FMR), and phase angle (PhA). Measurements were taken before and after the 24-session intervention period. Within-group comparison was done using paired *t*-test and Wilcoxon signed rank test. Between group comparison was done with one-way Anova or Kruskal Wallis tests by using mean differences for each group.

**Results:**

Paired *t*-test results revealed significant increment in VO_2_ peak (p=.006), handgrip strength (p=.037), 30-s chair stand test (p=.004) for the AE group. Additionally, Paired *t*-tests results revealed a significant reduction in fat percentage (p=.019) and increment in hand grip strength (p = 0.019), 30-s chair stand test (p = 0.010), and PhA (p=.029) for the Activ4Brain group. Additionally, although no significant differences were observed in the within-group comparisons for the TUG test in any of the groups, the changes differed significantly between the control and aerobic exercise (AE) groups (p = .033), as indicated by the Kruskal–Wallis test.

**Conclusion:**

Both the AE and Activ4Brain interventions resulted in improvements in physical fitness suggesting that the inclusion of cognitive training in the Activ4Brain program did not compromise its ability to also improve physical function. Moreover, the Activ4Brain program also resulted in improvements in body composition by reducing fat percentage and increasing PhA. The Activ4Brain program was a useful strategy to improve physical fitness and maintain body composition in older persons, while also incorporating cognitive training.

## 1 Introduction

As longevity is increasing, one of the main concerns is the increment in dementia rates with aging (Gianfredi et al., 2025). While exercise is highlighted as an effective strategy against neurodegenerative disorders, simultaneous integration of exercise and cognitive training has been shown to have additional benefits on cognitive function, especially when performed in a social context (Rieker et al., 2022). These integrated programs apply cognitive training while participants perform exercise. Integration of cognitive training with exercise can improve motor automaticity by increasing connectivity between the cerebellum and the motor cortex (Xiao et al., 2023). While exercise facilitates neuroplasticity by increasing neurotrophic factors such as brain-derived neurotrophic factor (BDNF) and insulin like growth factor (IGF-1) (Behrad et al., 2024), cognitive training strengthens the connections between different brain regions (Chen and Hao, 2025). Enhanced neuroplasticity and motor automaticity not only improve cognitive function but also facilitate adaptation to movement demands, such as postural control and balance (Yadolahi et al., 2024; Divandari et al., 2026) which eventually benefits physical functioning. Indeed dual-task training has been shown to improve balance (Ali et al., 2022) and functional mobility (Yu et al., 2024). Furthermore, dual task training has the potential to enhance physical performance through automatic and conscious processing (Yu et al., 2024).

Notably, the simultaneous integration of exercise and cognitive training can yield effects on physical fitness components comparable to those achieved through exercise alone (Gavelin et al., 2021). Thus, this approach could be an effective strategy to maximize cognitive benefits while also improving physical fitness and body composition (Gajewski et al., 2023).

A possible approach to develop an integrated exercise program, is integrating aerobic exercise with cognitive training. This integration can enhance both physical fitness and cognitive function, promoting overall health (Gavelin et al., 2021). A recent scooping review suggested that moderate continuous aerobic exercise should be incorporated into dual task training due to its potential to increase cardiorespiratory fitness in older persons (Petrigna et al., 2025). Additionally, walking ability under dual-task conditions decreases with aging which negatively affects activities of daily living, and dual task training that incorporates walking can positively impact balance and walking speed that eventually improve daily life activities and reduce fall risks in older persons (Varela-Vásquez et al., 2020). Aerobic exercises are defined as rhythmic bodily movements designed to improve cardiorespiratory fitness (Millstein, 2013). Moreover, aerobic exercises contribute to preserving cognitive function by decreasing inflammation, and increasing neurotrophic factors and angiogenesis, in older people (Akalp et al., 2024). Furthermore, aerobic fitness, leg strength and mobility decline with aging and aerobic exercises are effective in reversing this tendency in older persons (Brightwell et al., 2019). Thus, aerobic exercises can increase the quality of life and decrease morbidity by increasing physical fitness(Bouaziz et al., 2017).

In older persons, aerobic exercise interventions have been shown to improve body composition by decreasing fat mass and increasing muscle mass (Konopka and Harber, 2014). Thus, aerobic exercise can reduce Fat to Muscle Ratio (FMR). FMR is calculated by dividing fat mass by muscle mass. FMR has been suggested to be a better tool to detect fat change than body mass index (BMI) (Lafontant et al., 2023). Elevated fat mass increases inflammation (Khanna et al., 2022) which may eventually lead to the development of chronic diseases (Santanasto et al., 2016). Kim and colleagues also suggested that higher muscle mass and quality are linked with better metabolic health (Kim et al., 2021). The term muscle quality refers to the strength or power generated by per unit of muscle (Barbat-Artigas et al., 2012; Naimo et al., 2021) and phase angle can be a predictor of muscle quality (Geng et al., 2022). The phase angle reflects cellular health and decreases with aging, and has been associated with physical fitness and muscle strength (Yamada et al., 2022). Moreover, phase angle has been shown to be associated with global cognitive function measured with the Mini-Mental State Examination test in older persons (Zeng et al., 2025). In addition to exercise, cognitive training, especially training of executive functions, may affect health related behaviours through self-regulation, and reduce body weight in the long term (Szabo-Reed and Donnelly, 2021). Thus, simultaneous integration of cognitive training with aerobic exercise might not only affect cognition but also have additional benefits on body composition and physical fitness.

Despite aerobic exercise being recommended to maintain physical functioning in older persons (Nelson et al., 2007), to the best of our knowledge, the synergist effects of integrating cognitive exercises with aerobic training on physical fitness and body composition have not been comprehensively investigated. Moreover, effectiveness of the intervention may vary depending on the method of integration. Indeed, a recent meta-analysis demonstrated that when integration of cognitive training with exercise is done simultaneously and in a social context, it may increase the effectiveness of the program on physical function and cognitive outcomes (Rieker et al., 2022).

To address these aspects, we developed the Activ4Brain program, which integrates cognitive training (targeting cognitive flexibility, inhibitory control, attention, and working memory) into a moderate-intensity group aerobic exercise program. The Activ4Brain program simultaneously combines cognitive training with moderate-intensity aerobic exercise in a social context.

Thus, the primary aim of the current study is a) to evaluate the effects of the Activ4Brain program on physical fitness (aerobic fitness, muscle strength and mobility) and on body composition (fat mass, fat percentage, muscle mass, FMR, phase angle). The secondary aim of the study is to ensure that the Active4Brain program is as effective as an aerobic exercise program alone containing the same rhythmic bodily movements, on body composition and physical fitness. Given the potential of simultaneously integrated exercise programs to improve cognitive function and physical fitness (Rieker et al., 2022), understanding their synergist effects on physical outcomes will facilitate the development of targeted exercise programs. Furthermore, this study will address the applicability of the Activ4Brain program as a group aerobic exercise modality for older persons.

## 2 Methods

### 2.1 Study design

This study is part of a three arm, parallel design, quasi experimental controlled trial. Participants allocation to the groups was performed by a researcher in a quasirandom manner to ensure that the groups were balanced regarding sex and age. In some cases, allocation to the groups was done by convenience to ensure that couples would be assigned to the same group (facilitating participation). The 3 balanced groups were then randomly allocated to each of the experimental and control groups. The trial was registered at the Clinicaltrials.gov (NCY07108413). The study was approved by the Ethical Committee of the Faculty of Sports Science and Physical Education, University of Coimbra, Portugal with the reference number CE/FCDEF-UC/00082023. All procedures were performed according to Helsinki Declaration.

### 2.2 Participants

Individuals aged 55–75 years were recruited using flyers, newspaper advertisement and social media in the Coimbra region, Portugal. The interventions lasted between January 2024 and April 2025. The inclusion criteria were a) being between 55 to 75 years old and b) being able to perform exercise according to the Physical Activity Readiness Questionnaire (PAR-Q). The exclusion criteria were: a) severe depression according to Geriatric Depression Scale (score above 20); b) having a clinical diagnostic of any neurodegenerative disease; c) having cognitive impairment as assessed by the Portuguese version of the Addenbrooke´s Cognitive Examination-Revised; d) having a high falls risk assessed by the timed up and go test and the chair stand test.

**FIGURE 1:**
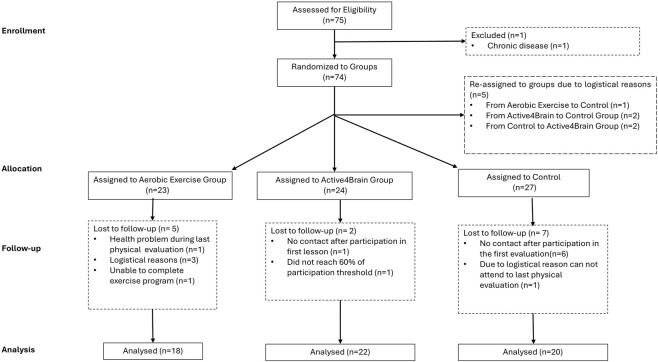
The Study flow diagram indicates each intervention step (enrolment, allocation, follow-up and data analysis).

The sample of this study was drawn from a larger project. The original sample size was determined according to primary end point of cognitive function which will be reported elsewhere. Detailed description regarding to participant allocation, sample size calculation and study design were reported in our study protocol (Besikci et al., 2026). Seventy-four participants met our eligibility criteria. The participants were divided into three groups, matched for age and sex ratios. Subsequently, matched groups were randomly assigned to the control group (CG), aerobic exercise group (AEG) and Activ4Brain group (Activ4BrainG) (Figure 1). However, due to logistic reasons, we re-assigned a total 5 participants (2 participants from the CG were moved to the Activ4BrainG, 2 participants from the Activ4BrainG were moved to the CG and one moved from the AEG to the CG). A written informed consent form was signed by the participants before the start of the study. Physical activity (PA) levels were measured using the Portuguese version of the International Physical Activity Questionnaire (IPAQ) and the level of PA was classified as low, moderate and high level (Table 1) (Sardinha et al., 2010). Baseline medication usage was documented. Participants were instructed to report any change in their medical status, medications or lifestyle habits including physical activity and in any specific diet. In case of changes in medical status participants were removed from analysis. Maintenance of physical activity levels, diet and medication usage were verified during exit interviews that were conducted by certified psychologists.

**TABLE 1:** Baseline demographics, physical activity status, and medical characteristics by groups.

Variables (N = 74)	CG (N = 27)	AEG (N = 23)	Activ4BrainG (N = 24)	P- value
Age (years)	65.7 ± 5.54	66.3 ± 6.5	64.5 ± 6.9	0.637
Sex (Female/Male)	16/11	13/10	12/12	0.795 ( $\left.\chi^{2}\right)$
Low level physical activity	3	3	2	0.529 ( $\left.\chi^{2}\right)$
Moderate level physical activity	18	10	14	
High level physical activity	6	10	8	
Thyroid diseases	5	1	3	0.419 ( $\left.\chi^{2}\right)$
Type 2 diabetes	2	3	-	
Pulmonary emphysema	-	1	-	
High cholesterol	12	7	9	
Hypertension	10	6	4	
Osteoarthiritis	-	3	1	
Arrhytmia	1	-	1	

The categoric variables are presented as numbers and the continuous variables are presented as mean and standard deviation. Comparison of continuous variables in between groups was done with One-way ANOVA. Comparison of categorical variables was done with Chi-Squared Test( $\chi^{2}$ ).

During the evaluations, evaluators were not blinded due to the fact that the evaluations were performed by the same researchers that were involved in the interventions (K.A. and H.B).

### 2.3 Intervention

The AE and Activ4Brain programs consisted of 24 sessions, twice a week. Each session had a duration of 50 min and consisted of 5 min warm-up, 40 min main session and 5 min cool down periods. AE and Activ4Brain programs were group based aerobic exercise programs. Exercise intensity was controlled using Polar Team Pro system (Polar Team Pro, Polar Electro, Kempele, Finland) enabling the simultaneous tracking of multiple participants heart rate (HR). Intensity for the AE and Activ4Brain programs was set between 60%–80% of Maximal HR calculated using Tanaka´s Formula (HR_max_ = 208-(0.7 x age) (Tanaka et al., 2001).

The AE program consisted of 4 blocks of 5 min each. Each block consisted of rhythmic whole-body movements requiring simultaneous coordination of upper and lower limbs. When the participants completed the 4^th^ block, they repeated all blocks again and completed 40 min of the main training session. A detailed description of the organised blocks is presented in Supplementary Table S1.

The Activ4Brain program incorporated cognitive training in the aerobic exercise program. The program consisted of 4 blocks of 10 min each. In each block, participants performed 5 min of rhythmic bodily movements (the same as the AE exercise group) and subsequently performed cognitive training for 5 min while marching in place. Responses to the cognitive tasks were executed via four color-coded (white, green, yellow and blue) and numbered (1,2,3,4) pedals. The cognitive training tasks targeted the following cognitive domains: attention, inhibitory control, processing speed, working memory and decision-making. Marching in place during the cognitive training aimed at maintaining cardiovascular activation throughout.

### 2.4 Assessment of physical fitness and body composition

Physical fitness was assessed by handgrip strength, timed up and go (TUG), 30-s chair stand test and a submaximal cardiopulmonary exercise test (CPET). All physical evaluations were performed in the morning, for 2 weeks before and after the intervention.

Stature was measured using a stadiometer (Seca Bodymeter, Model 208, Germany). Body composition was evaluated through bioimpedance (InBody 770; Biospace Co.) and determined total body weight, fat mass, fat percentage, muscle mass and the phase angle. Additionally, fat to muscle ratio (FMR) was calculated by dividing fat mass by muscle mass values obtained using bioimpedance data.

Handgrip strength was measured using a hydraulic hand dynamometer (Lafayette, Model 5030L1, United States) while participants were standing with the dominant arm fully extended. The handle size was adjusted according to the participants hand size and participants were instructed to squeeze the handle with maximum effort. Effort continued for 5 s with 3 attempts. The rest time between attempts was 30 s. The best value of the attempts was recorded in kilogram-force (Lemmink et al., 2001).

In order to evaluate falls risk and eligibility for our exercise program, we applied the Timed UP and GO (1991) and 30-Second Chair Stand (2017). Participants started TUG in a sitting position on a standard chair and were instructed to stand, walk to the marker 3 m away, walk back to the chair at their normal pace and sit down again. Time was recorded with a stopwatch. Participants began the 30-s chair stand test from a seated position on a chair and were instructed to cross their arms over their shoulders, rise to the standing position and sit back down again for 30 s. The total number of repetitions was recorded. If participants were more than halfway to sitting, when time elapsed, that cycle counted as a repetition.

CPET was conducted using a submaximal incremental test on a cycle ergometer (Lode-Excalibur, Netherlands). CPET started with an initial load of 50 W for female and 75 W for male participants. The resistance was increased every 3-min and the test continued at 60–70 rpm cadence (Noonan and Dean, 2000). CPET stopped when participants reached 85% of their maximal HR calculated using Tanaka’s Formula (Tanaka et al., 2001). Gas exchange measurement was performed using breath-by-breath analysis with The Quark CPET system (COSMED-the Metabolic Company, Rome, Italy). Data was averaged for analysis using 30 s periods with the software OMNIA (v1.6.5.). The gas exchange measurement system was calibrated daily, and a turbine (28 mm) calibration was done before each test according to the manufacturer’s instructions.

VO_2_ Peak was calculated as the average VO_2_ of the last 30 s of the CPET. First Ventilatory Threshold (VT) was determined using the V-slope method (Beaver et al., 1986). Relative VO_2_ Peak and VT were reported as millilitres per kilogram of body weight per minute.

Feasibility measurements were done using adherence percentage, adverse events, and dropouts. Adherence percentage and adverse events occurrence were recorded by two researchers during each exercise session (K.A. and H.B.).

### 2.5 Statistical analysis

Descriptive statistics are reported as means and standard deviations (SD) for continuous variables, while categorical variables are presented as counts and percentages. Assumption of normality was checked using the Shapiro-wilk test with a significance threshold of 0.05 and Skewness and Kurtosis values were assessed within −2 and +2 range (George and Mallery, 2010). VT scores in control group and AE, BMI in AE, Fat mass in AE and Activ4Brain group violated assumption of normality. Additionally, difference scores in VT, chair stand, TUG, BMI, Fat percentage and muscle mass data violated the assumption of normality. Therefore, we used non-parametric tests to compare those variables. To provide normal distribution, the TUG and chair stand data were converted into a logarithmic scale. Assumption of homogeneity was checked using Leven`s Test with a significance threshold of 0.05 and Group differences were analysed using one-way ANOVA for normally distributed variables, the Kruskal Wallis Test for non-normally distributed variables and the chi-squared test for categorical variables. Additionally, between groups comparisons were done using differences between time points (post intervention values (M2) – baseline values (M1)). Subsequently, differences between groups were analysed using either one-way ANOVA or the Kruskal Wallis test, depending on whether data met the assumptions of normality and homogeneity. In case significant differences in changes between groups were present, Bonferroni *post hoc* test was applied for one-way Anova and Dunn´s test for the Kruskal Wallis Test results. Within-group comparisons between the pre-intervention (baseline) and post-intervention (follow-up) were conducted using paired *t*-test for normally distributed variables and Wilcoxon signed rank test for non-normal distribution. The magnitude of difference between time points (Effect Sizes:ES) were calculated according to Cohen´s *d* and classified as very small ES (*d* < 0.2), small ES (0,2 < *d* < 0.5), Medium (0.05 < d < 0.8) and large ES (*d* ≥ 0.8) (Cohen, 1988). For between group comparison, effect sizes were calculated as eta-squared (η^2^) for one-way Anova and for Kruskal Wallis test (η_H_^2^). Interoperation of effect sizes (ES) were done by using the following cut of points: very small ES (η^2^ < 0.01), small ES (0.01<η^2^ < 0.06), medium ES (0.06< η^2^ < 0.14) and large ES (η^2^ > 0.14) (Cohen, 2008). Additionally, Significance value set as 0.05 and all statistical analysis were performed using IBM SPSS Statistics version 30.0. Graphs were created using GraphPad Prism version 9.0.

## 3 Results

### 3.1 Drop out, adherence rate, and adverse events

Sixty-one (out of 74) participants completed the 24 intervention sessions and participated in the follow-up evaluation. Thirteen participants did not complete the intervention and one participant from the AEG was not able to participate in the last physical evaluation due to health-related concerns (Figure 1). Adherence rate (percentage of sessions attended) was 86% ± 12% for the AEG, corresponding to an average (mean ± SD) of 21 ± 3 sessions per participant and 90% ± 8% corresponding to an average (mean ± SD) of 22 ± 2 sessions per participant for the Activ4BrainG. Moreover, five participants in the Activ4brainG and two participants in the AEG completed all the 24 intervention sessions. The intensity was average HR during the sessions were 69% for the AEG and 68% for the Activ4Brain group.

During the exercise programs no major adverse events occurred. Three participants from the AEG and one participant from the CG did not complete CPET at the last evaluation due to expression of health-related concerns not related to the intervention. Baseline and follow-up values of the participants are reported for each group in Table 2.

**TABLE 2:** Physical fitness and body composition baseline and post intervention values from the participants who completed the intervention.

​	Control					Aerobic exercise					Activ4Brain					Between group comparison	
Physical fitness	N	Before	After	M2-M1	p-value	N	Before	After	M2-M1	p-value	N	Before	After	M2-M1	p-value	F/H value	p-value
VO_2_ peak (mL/min/kg)	19	20.97 ± 3.37	22.09 ± 3.15	1.13 ± 2.64	0.079	15	24.27 ± 4.13	26.51 ± 5.82	2.24 ± 2.70	0.006**	22	24.57 ± 6.59	26.03 ± 5.16	1.46 ± 4.39	0.133	F = 0.448	0.641
VT1 (mL/min/kg)	19	15.45 ± 2.64	16.05 ± 2.69	0.6 ± 3.19	0.231^a^	15	17.25 ± 2.64	18.1 ± 3.12	0.85 ± 3.06	0.306^a^	22	17.28 ± 3.45	17.68 ± 3.19	0.4 ± 2.75	0.503	H = 0.264	0.876
Hand grip strengtd (kgf)	20	35.85 ± 11.62	37.8 ± 13.13	1.95 ± 4.17	0.0501	17	34.47 ± 11.02	36.59 ± 9.55	2.12 ± 3.84	0.037*	22	34.81 ± 14.10	37.18 ± 12.00	2.36 ± 4.36	0.019*	F = 0.053	0.949
Chair stand (repetitions)^log^	20	18.35 ± 4.02	18.9 ± 4.89	0.55 ± 4.27	0.655^log^	17	19 ± 3.60	20.88 ± 4.10	1.88 ± 2.34	0.004**^log^	22	16.95 ± 3.61	19.36 ± 4.74	2.41 ± 4.73	0.010*^log^	H = 1.173	0.556
TUG (Seconds)^log^	19	6.78 ± 1.15	7.12 ± 1.03	0.34 ± 1.04	0.117^log^	17	6.46 ± 1.34	6.23 ± 1.33	−0.23 ± 0.75^a^ ^-c^	0.271^log^	22	6.48 ± 1.06	6.44 ± 0.84	−0.04 ± 0.83	0.821^log^	H = 6.830	0.033*

Body composition	N	Before	After	M2-M1	p-value	N	Before	After	M2-M1	p-value	N	Before	After	M2-M1	p-value	F/H value	p-value
BMI	20	26.23 ± 4.08	26.17 ± 4.06	−0.06 ± 0.71	0.712	18	25.47 ± 4.61	25.41 ± 4.43	−0.07 ± 0.59	0.74^a^	22	26.5 ± 3.60	26.38 ± 3.29	−0.12 ± 1.22	0.653	H = 1.105	0.575
Fat Mass (kg)	20	23.95 ± 7.84	23.75 ± 7.73	−0.20 ± 1.89	0.649	18	21.36 ± 9.80	21.06 ± 9.56	−0.30 ± 1.56	0.601^a^	22	23.89 ± 7.45	22.87 ± 6.87	−1.02 ± 2.04	0.06^a^	F = 1.231	0.3
Fat percentage (%)	20	32.44 ± 6.58	32.08 ± 6.97	−0.36 ± 2.15	0.471	18	29.93 ± 7.75	29.57 ± 7.90	−0.36 ± 1.87	0.432	22	33.6 ± 7.14	32.3 ± 6.52	−1.30 ± 2.42	0.019*	H = 1.142	0.565
Muscle Mass (kg)	20	26.84 ± 6.43	26.94 ± 6.23	0.10 ± 1.10	0.704	18	26.37 ± 4.79	26.49 ± 4.69	0.12 ± 0.79	0.52	22	25.91 ± 6.54	25.85 ± 6.44	−0.06 ± 2.18	0.893	H = 0.446	0.8
FMR (fat Mass/Muscle Mass)	20	0.91 ± 0.27	0.9 ± 0.27	−0.01 ± 0.089	0.586	18	0.81 ± 0.31	0.8 ± 0.30	−0.01 ± 0.07	0.466	22	0.96 ± 0.32	0.92 ± 0.29	−0.04 ± 0.11	0.091	F = 0.709	0.496
Phase angle (°)	20	5.11 ± 0.69	5.06 ± 0.70	−0.06 ± 0.25	0.346	18	5.13 ± 0.59	5.22 ± 0.57	0.09 ± 0.21	0.088	22	5.17 ± 0.71	5.27 ± 0.65	0.10 ± 0.19	0.029*	F = 3.025	0.056

N: number of participants.

^a^ the value obtained from Wilcoxon Signed Rank Test; F: F-value obtained from the One-way ANOVA, test; H: H - value obtained from the Kruskal Wallis Test.

^log^: logarithmically transformed data.

^a-c^: significant difference in changes between aerobic exercise and control group; TUG: timed up and go test; kgf: kilogram force; BMI: body mass index; FMR: fat to muscle ratio; kg: kilogram; *: p < 0.05; **:p<.

### 3.2 Intervention effects on physical fitness

**FIGURE 2:**
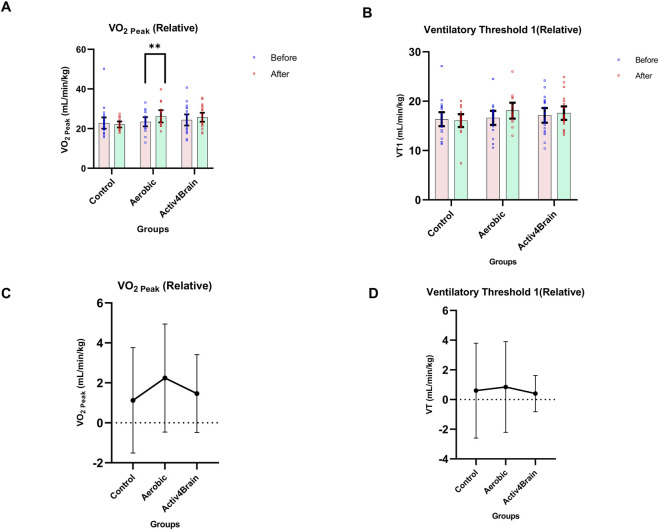
Changes in Cardiorespiratory Fitness after 24 intervention sessions of in CG, AEG and Active4BrainG; **(A)** Changes in VO_2 Peak_; **(B)** Changes in the First Ventilatory Threshold; **(C)** Comparison of Group changes in VO_2 Peak_; **(D)** Comparison of Group Changes in Ventilatory Threshold. *P* values from paired samples *t*-tests. ** = p < 0.01. Error bars were created by using means and 95% CI.

The physical fitness assessment consisted of VO_2_ peak, VT1, handgrip strength, 30 s chair stand test and TUG. Paired sample t-tests revealed significant increment at VO_2_ peak values in AEG (t(14) = 3.207; 0.742 to 3.737 95% CI; *d* = .823 (large effect); p=.006) while there were no significant changes in the CG (t(18) = 1.860;-0.146 to 2.398 95%CI; *d* = .427 (small effect); p=.079) or the Activ4BrainG (t(21) = 1.562;-0.485 to 3.412 95%CI; *d* = .333 (small effect); p=.133). However, these changes were not statistically significant across groups (F(2,53) = .448; p=.641, η^2^ = 0.17 (large effect)) (Figure 2). Additionally, no significant statistical differences were found within CG (z = −1.198, p=.231, r = 0.28 (small effect)) and AEG (z = −1.023, p=.306, r = 0.26 (small effect)) and across groups (H(2) = .264; p=.876, η_H_^2^ = −0.03 (zero effect) in the VT (Figure 2).

Paired samples *t*-tests revealed a significant increment on handgrip strength in the AEG (t(16) = 2.275; 0.14 to 4.09 95% CI; *d* = .552 (medium effect); p=.037) and in the Activ4BrainG (t(21) = 2.54; 0.434 to 0.30 95% CI; *d*=.542 (medium effect); p=.019) but not in the CG (t(19) = 2.090;-0.003 to 3.903 95%CI; *d* = .467 (small effect); p=.0501). However, those changes were not statistically significant across groups (F(2,56) = 0.053, p=.949, η^2^ = 0.002 (very small effect)).

The Chair stand test and TUG test values were transformed to logarithmic scale to provide a normal distribution and the use of parametric statistical tests. Non-transformed mean and standard deviation values are shown in Table 2. Paired samples *t*-tests revealed significant increment at 30-s chairs stand test in AE (t(16) = 3.415; 0.016 to 0.066 95% CI; *d* = .828 (large effect); p=.004) and Activ4Brain (t(21) = 2.848; 0.015 to 0.09 95% CI; *d*=.607 (medium effect); p=.010). However, changes were not statistically significant in the CG (t(19) = 0.454; −0.036 to 0.056 95% CI; *d* = .102(very small effect); p=.655) and across groups (H (2) = 1.173, p=.556, η_H_^2^ = −0.02 (zero effect)).

**FIGURE 3:**
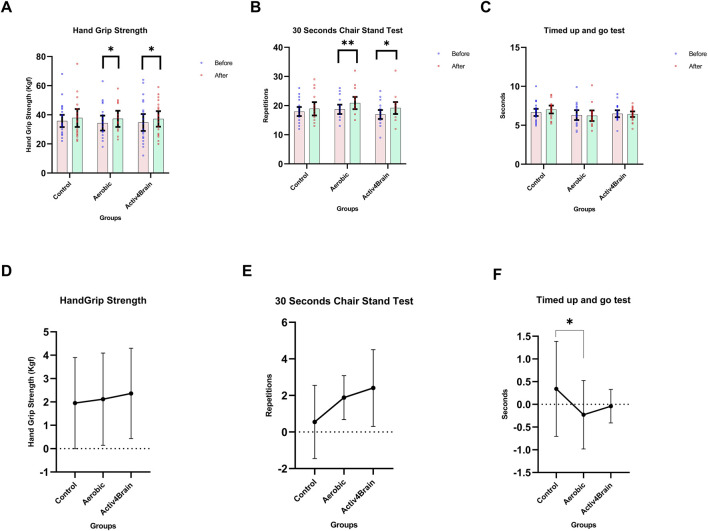
Changes in Handgrip Strength, 30-s Chair Stand Test and Timed Up and Go Test after 24 sessions of intervention period in CG, AEG and Active4BrainG; **(A)** Changes in Handgrip Strength; **(B)** Changes in 30-s Chair Stand Test; **(C)** Changes in Timed Up and Go Test; **(D)** Comparison of Group Changes in Handgrip Strength; **(E) **Comparison of Group Changes in 30-s Chair Stand Test; **(F)** Comparison of Group Changes in Timed Up and Go Test. P values from paired samples t-tests and Kruskal Wallis Test. * = p < 0.05, ** = p < 0.01. Error bars were created by using means and 95% CI.

Paired samples *t-*tests showed no significant statistical changes within groups in TUG scores (AEG (t (16) = -1.141; −0.044. to 0.013 95% CI; *d* = -.277(small effect); p=.271); Activ4BrainG (t (21) = -.085; −0.025. to 0.023 95% CI; *d* = -.18(very small effect); p=.933) CG (t(18) = 1.647; −0.006. to 0.051 95% CI; *d* = .378 (small effect); p=.117)). However, Kruskal Wallis Test revealed significant statistical differences between groups (H(2) = 6.830, p=.033, η_H_^2^ = 0.09(medium effect)) in the TUG test. Pairwise comparison of changes in means revealed a significant difference between AEG and CG (p=.036, z = 2.52, r = 0.41 (medium effect)) (Figure 3).

### 3.3 Intervention effects on body composition

**FIGURE 4:**
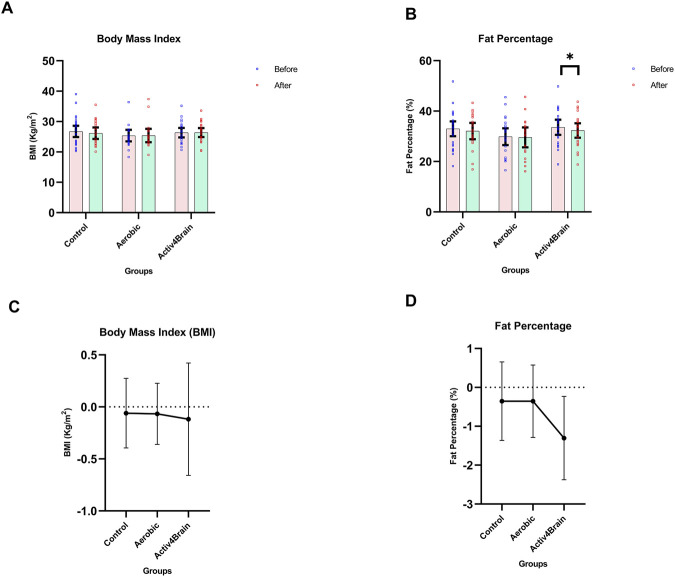
Changes in Body Mass Index and Fat Percentage after 24 sessions of intervention in CG, AEG and Active4BrainG; **(A)** Changes in Body Mass Index; **(B)** Changes in Fat percentage; **(C)** Comparison of Group Changes in Body Mass Index; **(D)** Comparison of Group Changes in Fat Percentage. Error bars were created by using means and 95% CI.

**FIGURE 5:**
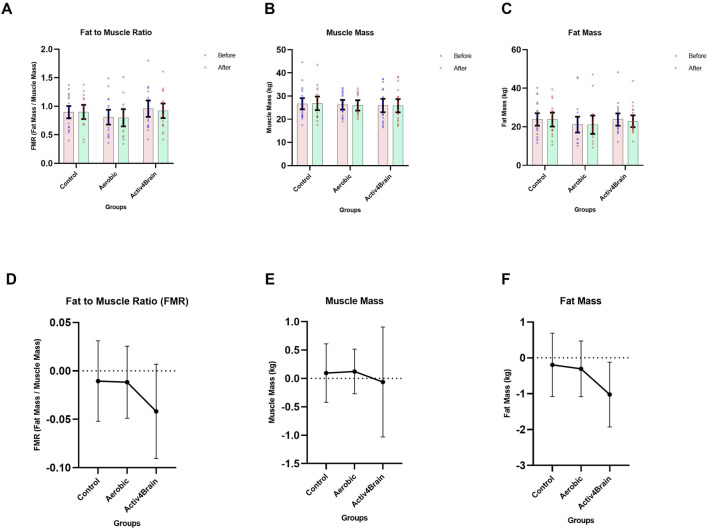
Changes in Fat to Muscle Ratio, Fat Mass and Muscle Mass after 24 sessions of intervention in CG, AEG and Active4BrainG; **(A)** Changes in Fat to Muscle Ratio; **(B)** Changes in Muscle Mass; **(C)** Changes in Fat Mass; **(D)** Comparison of Group changes in Body Mass Index; **(E)** Comparison of Group Changes in Muscle Mass; **(F)** Comparison of Group Changes in Fat Mass. P values from paired samples t-tests. * = p < 0.05, Error bars were created by using means and 95% CI.

**FIGURE 6:**
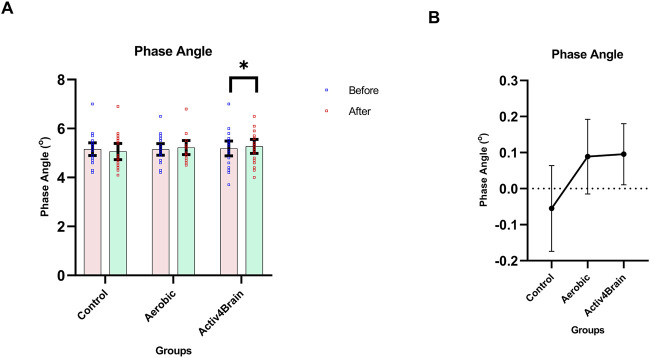
Changes in Phase Angle after 24 sessions of intervention in CG, AEG and Active4BrainG; **(A)** Changes in Phase Angle; **(B)** Comparison of Group Changes in Phase Angle. P values from paired samples t-tests. * = p < 0.05. Error bars were created by using means and 95% CI.

Body composition analysis includes BMI, fat percentage (Figure 4), fat mass, muscle mass, FMR (Figure 5) and phase angle (Figure 6) variables. Fat percentage was significantly reduced in Activ4BrainG (paired samples *t*-test (t(21) = −2.531; −2.376 to −0.233 95%CI; *d* = -.540 (medium effect); p=.019) while no significant changes were observed in CG (t(19) = -0.736; −1.364 to 0.654 95% CI; *d* = -.165 (very small effect); p=.471) and in the AEG (t(17) = -0.805; −1.287 to 0.576 95% CI; *d* = -.653 (medium effect); p=.432). Additionally, phase angle significantly increased in the Activ4BrainG (paired samples *t*-test t(21) = 2.339; 0.011 to 0.18095%CI; *d* = .499(small effect); p=.029) while no significant changes occurred observed in CG (t(16) = -0.967; −0.174 to 0.064 95% CI; *d* = -.216(small effect); p=.346) and AEG (t(17) = 1.810; −0.014 to 0.192 95% CI; *d* = .427(small effect); p=.088). Both changes in fat percentage (H(2) = 1.142, p=.56, η_H_^2^ = −0.02) and phase angle (F(2.57) = 3.025, p=.056, η^2^ = 0.096(medium effect)) were not statistically significant across groups. Moreover, no significant differences were observed within and between groups in BMI (H(2) = 1.105, p=.575, η_H_^2^ = −0.02), fat mass (F(2,57) = 1.231, p=.30, η^2^ = 0.041(small effect)), muscle mass (H(2) = 0.446, p=.80, η_H_^2^ = −0.03(zero effect)) and FMR (F(2,57)=.709, p=.496. η^2^ = 0.024(small effect)).

## 4 Discussion

Given the potential of simultaneously integrated exercise programs to improve cognitive function and physical fitness, this study aimed to determine whether the Activ4Brain program was as efficient as the aerobic-only program on improving body composition and physical fitness The results revealed a significant reduction in Fat percentage (medium ES: *d* = -.540) and an increment in phase angle (small to medium ES: *d* = .499) in the Activ4BrainG while there were no changes in AEG and CG. Additionally, significant improvements were observed in handgrip strength and 30 s chair stand test in both AEG and Activ4BrainG, while VO_2_ Peak had a significant increase in the AEG (Large ES: *d* = .823). Furthermore, high adherence rate to exercise programs indicates feasibility and acceptability of both AEG and Activ4Brain exercise programs, suggesting both exercise programs are well-tolerated.

### 4.1 Physical fitness

Physical fitness is a critical determinant of health and wellbeing, as improvements in physical fitness offer protective benefits against comorbidities and all-cause mortality across all age groups including older persons (Äijö et al., 2016; Bull et al., 2020). Physical fitness components tend to decrease with age. Moreover, physical improvements in aerobic fitness, mobility, muscle strength, and endurance are associated with lower risk for dementia (Boyle et al., 2009; Ahlskog et al., 2011; Serna Orozco et al., 2025). Several studies investigated the dual-task training effects on physical function and suggested that dual-task training can enhance physical fitness parameters such as endurance, functional mobility and balance (Li et al., 2023; Yu et al., 2023; Díaz-García et al., 2025). Moreover, these improvements may originate from the synergy between cognitive training and exercise as a result of the increment in mental fatigue resilience which may contribute to exercise tolerance (Díaz-García et al., 2025). Finally, improvement in the communication between sensorimotor and cognitive control may improve functional mobility in older persons (Li et al., 2023).

One of the limitations of previous studies which found significant improvements in cardiorespiratory endurance performance was that they utilized indirect measurements such as the 6-min walking test (Jardim et al., 2021), or the 10-min walking test (Hwang et al., 2021). In our study, we utilized direct cardiorespiratory measurements with cycle ergometry. The results showed that the Activ4brain program did not improve cardiorespiratory fitness (VO_2_ peak and VT1) as effectively as the AE program. First plausible explanation is that, we used a standardized intensity training program in which exercise intensity was balanced between groups, similar intensity can result in different physiological stress levels that eventually lead to different adaptations across different older persons (Katch et al., 1978). Indeed, Fabre and colleagues demonstrated that individualized intensity exercise program is more effective in improving aerobic fitness than standardized intensity program in older persons (Fabre et al., 1997). However, due to the fact that our intervention program was implemented in group classes, using individualised exercise intensity (e.g., based on Ventilatory threshold) was not feasible. Secondly, these results may have originated from the interruption in the AE blocks with cognitive training in the Activ4Brain program. The cognitive training part of the exercise which lasted a total of 20 min, was performed while marching in place and involved only lower body movements to respond to the cognitive tasks. This may have reduced the physiological effort and disrupted the rhythmic movements leading to reduced physiological adaptations that increase aerobic fitness compared to the AEG. Indeed, additional upper body use may affect the development of aerobic fitness (Marterer et al., 2023). Therefore, future research should investigate the importance of including both lower and upper body exercises when developing combination strategies of cognitive training and exercise to maximize benefits related to aerobic fitness.

No significant changes were observed in VT across groups. A plausible explanation is that, although exercise intensity and duration were appropriate for the development of first VT, the majority of the participants were already physically active as assessed through the IPAQ questionnaire (N = 8 low, N = 42 moderate and N = 24 high level physically active) therefore, our standardized exercise program may have under or over stimulated individuals cardiorespiratory adaptations that affect VT that eventually led to negative results (Fabre et al., 1997). Further research should carefully adjust exercise intensity to combined exercise programs (with cognitive training) to maximise cardiorespiratory adaptations.

Our study demonstrated that the Activ4Brain program (combining AE with cognitive training) was as effective as a complete session of aerobic exercise on improving handgrip strength and leg muscle endurance. Handgrip strength and 30 s chair stand tests results of Activ4Brain program were parallel with those of a systematic review that measured combined physical exercise and cognitive training effects on strength (Yu et al., 2023). Moreover, moderate aerobic exercise enhances muscle quality and quadriceps strength by increasing capillary density and myofibrillar protein synthesis in older persons (Brightwell et al., 2019). Thus, enhanced muscle quality may improve handgrip strength and 30 s chair test results similarly in both AEG and Activ4BrainG.

Our results demonstrated that exercise programs did not significantly affect TUG results. TUG measures functional mobility, balance and fall risk (Benavent-Caballer et al., 2016). Our participants had average baseline scores of 6.50 ± 1.17 s, which indicates the participants had high mobility with no risk of fall (Gatenio-Hefling et al., 2025). The fact that at baseline the participants already had a good TUG score might have hindered any improvements. As participants showed good motor automaticity during dual-task exercise at baseline that might have reduced the impact of cognitive training on physical functioning (Yu et al., 2024). In this case, cognitive training may not lead to enhanced coordination by improving motor automaticity. Since the test was conducted at a normal walking rhythm, and it did not require extra effort, participants were already performing at ceiling performance at baseline and thus we did not observe any significant changes in TUG tests. Further studies should use more sensitive tests to assess combined exercise program effects (with cognitive training) on functional mobility.

### 4.2 Body composition

Association of body composition and cognitive function have been reported in the literature (Samuelsson et al., 2025). However, to the best of our knowledge dual-task training effects on body composition are not well understood. Kargaran and colleagues have showed that dual-task walking training with or without blood flow restriction reduced body mass, BMI and fat percentage. Moreover, dual-task walking training with blood flow restriction has yield more pronounced effects on body composition than dual-task training alone, indicating that the positive benefits of dual task training on body composition relies on increased energy expenditure during the exercise (Kargaran et al., 2021). We found a decrease in fat percentage and an increase in phase angle in the Activ4BrainG. A possible explanation for reduction in fat percentage is that cognitive demand may increase the glucose demand in the brain (Ampel et al., 2018) which eventually increases calorie deficit. Another possible explanation is that cognitive training, especially inhibitory control and mental flexibility, can affect daily habits and have the ability to improve body composition in the long term by improving diet and life style (Szabo-Reed and Donnelly, 2021). Indeed, the Activ4Brain program includes elements from cognitive flexibility and inhibitory control cognitive skills. Therefore, additional cognitive challenges with aerobic exercise may explain the significantly reduction in fat percentage for the Activ4BrainG. However, we did not monitor calorie deficit during the intervention, thus we can only speculate on indirect effects of the cognitive component of the Activ4brain program.

Phase angle reflects overall cellular health and muscle quality (Costa Pereira et al., 2024). Phase angle has been shown to be negatively associated with fat percentage (De Marco et al., 2025), however another study has demonstrated that phase angle is affected by small portions of body fat (Frau et al., 2025). Therefore, the combined effects of aerobic exercise on muscle quality (Brightwell et al., 2019) with the decrement in fat percentage could explain the significant improvement in phase angle seen in the Activ4BrainG. On the other hand, we did not observe significant changes in BMI, fat mass, muscle mass or FMR. However, there are many factors (e.g., diet, social and environmental factors), that could influence those variables and that were not controlled in our study (Feng, 2025). Therefore, the results regarding body composition should be interpreted carefully.

## 5 Practical implementation

In clinical and community perspectives, physical improvements with the Activ4Brain program are encouraging. Activ4Brain program yielded physical fitness benefits comparable to those of the AEG, aligning with existing literature (Gavelin et al., 2021). Furthermore, Activ4brain program offers a highly tolerable and feasible strategy for preserving muscle strength, improving body composition and enhancing cellular health in older adults. Notably, our combination method enables to perform cognitive training in a group exercise format. Moreover, in future implementations, it is possible to apply the Activ4brain program simultaneously to a higher number of participants (more than 4), thereby facilitating its large scale implementation and promoting social interaction while preserving the fundamental benefits of the intervention. Therefore, implementing the Activ4brain program offers a synergistic approach, successfully combining physical and cognitive demands to foster physical wellbeing and functional longevity through active aging. However, future developments of the program should consider the implications of the fact that the inclusion of cognitive tasks may disrupt rhythmic movement and overall physical exertion.

## 6 Limitations

This study has some limitations, the first one is that we used a standardized exercise intensity, and adaptations to exercise might differ among individuals (Fabre et al., 1997). Secondly, the study participants were physically active and had high mobility, thus significant changes in the TUG test and VT1 might not have been observed due to ceiling performance already at baseline. The fact that our participants were older people that were willing to participate in exercise programs may have resulted in selection bias of individuals that were physically active, and relatively healthy. Thus, our findings might not be generalised to highly sedentary older persons. Additionally, our exercise design, in terms of frequency, was below the ACSM standards which may also affect exercise adaptations and eventually the outcomes (Medicine et al., 2022). Another limitation was that diet, and environmental conditions were not controlled directly for in this study and therefore could have affected the changes in body composition (Feng, 2025). Another limitation was that although the study was planned as a randomized controlled trial, due to logistical reasons, a total of 5 participants were reassigned to different groups. Moreover, due to the fact that we used a sample size calculated for the main outcome (Besikci et al., 2026), our sample size is relatively small and this could have limited our ability to detect changes. Another limitation is duration of the intervention (24 sessions in 3 months). Although, the minimum duration suggested in the literature is 12 weeks (Li et al., 2023), for our exercise setting, a longer intervention period may be required to achieve exercise adaptations. In our study, we demonstrated mid-term effects of Activ4Brain program. A notable limitation of the present study is the lack of follow-up assessment to evaluate long-term sustainability of the observed effects on physical fitness, body composition and phase angle. Future studies should include follow up evaluation to determine long term efficacy, and sustainability of combined aerobic and cognitive training programs.

## 7 Conclusion

In conclusion, the Active4Brain program had positive but distinct effects on physical fitness and body composition. Regarding physical fitness, the results demonstrated that AE enhanced VO_2_ peak whereas the Activ4Brain program did not. However, the Activ4Brain program was as effective as the AE in improving upper body and lower body strength. In terms of body composition, the Activ4Brain program significantly decreased fat percentage and increased phase angle. This observation indicates that the Active4brain program had more pronounce effects on body composition than the AE program. However, these results must be interpreted considering certain methodological limitation. Overall our results indicate that the Active4Brain program is effective as an exercise program and preserves the benefits of an AE exercise only intervention, both in terms of strength and body composition. The Active4Brain program proved to be an effective, and holistic strategy. From a practical standpoint, since it requires minimal equipment and targets both physical and cognitive abilities, this program is highly suitable for real world implementation such as in senior day-care centres, sports centres and in preventive programs designed to prevent physical and cognitive decline in the aging population.

## Data Availability

The raw data supporting the conclusions of this article will be made available by the authors, upon justified request.
