# Supplementary material for: Efficacy of simultaneous aerobic and cognitive training (Activ4Brain) on physical fitness and body composition in older persons
Source: Front Aging. 2026 Jun 9;7:1819235. doi: 10.3389/fragi.2026.1819235 (PMC13287562; doi:10.3389/fragi.2026.1819235)
Supplement: Supplementary file 1 [file Table1.docx]

| Supplementary Table 1. Aerobic Exercise Program | | | | |
| --- | --- | --- | --- | --- |
| Exercises for 1 -4^th^ weeks | | | | |
|  | 20 seconds | 20 seconds | 20 seconds |  |
| 1.Block | V step | March | Step jack | 5 minutes |
| 2. Block | Arms and knee up | March | Box step | 5 Minutes |
| 3.Block | High Knee touch | March | Butt Kick | 5 Minutes |
| 4. Block | 4 steps forward and return with back walk | March | Sidestep (2 left-2 right) | 5 Minutes |
| Exercises for 5^th^ -9^th^ weeks | | | | |
| 1.Block | V step | March | Step Jack with butt kick | 5 minutes |
| 2. Block | Arms and knee up | March | Box step | 5 Minutes |
| 3.Block | High Knee Touches (with bounce) | March | Bounce sky punch | 5 Minutes |
| 4. Block | 4 steps forward and return with back walk | March | Sidestep ( 2 right-2 left with claps) | 5 Minutes |
| Exercises for 10^th^ -13^th^ week | | | | |
| 1.Block | V step | March | Bounce Sky Punch | 5 minutes |
| 2. Block | Arms and knees up | Clap benath the knee | Box step | 5 Minutes |
| 3.Block | Wide legs- bounce- touch the knee | March | Frontal Kick (with touching the toe) | 5 Minutes |
| 4. Block | 4 steps forward and return with back walk | March | Sidestep ( 2 right-2 left with claps) | 5 Minutes |
